# General Versus Regional Anesthesia for Emergency Cesarean Delivery in a High-volume High-resource Referral Center: A Retrospective Cohort Study

**DOI:** 10.2478/rjaic-2020-0012

**Published:** 2020-12-31

**Authors:** Kenas Wiskott, Raed Jebrin, Daniel Ioscovich, Sorina Grisaru-Granovsky, Aharon Tevet, Daniel Shatalin, Alexander Ioscovich

**Affiliations:** 1 Department of Anesthesiology, Perioperative Medicine and Pain Treatment, Shaare Zedek Medical Center, affiliated with Hebrew University, Jerusalem, Israel; 2 Department of Obstetrics & Gynecology, Shaare Zedek Medical Center, affiliated with Hebrew University, Jerusalem, Israel

**Keywords:** Emergency, cesarean delivery, regional anesthesia, general anesthesia

## Abstract

**Objective:**

The choice of anesthesia for emergency cesarean delivery (CD) is one of the most important choices to make in obstetric anesthesia. In this study, we examine which type of anesthesia was used for emergency CD in our hospital, and how the choice affected the time from entry to the operation room until incision (TTI), time until delivery (TTD), and maternal/neonatal outcomes.

**Methods:**

Retrospectively, we examined all emergency CD’s performed in Shaare Zedek Medical Center between January–December 2018. Results: 1059 patients met the inclusion criteria, of which 7.7% underwent general anesthesia (GA), 36.2% – conversion from labor epidural analgesia to surgical anesthesia, 52% – spinal anesthesia and 4.1% – combined spinal epidural. We did not find a significant difference between the GA and conversion epidural groups in terms of TTI or TTD. Nevertheless, GA was found to be correlated to a high rate of blood-products requirement and ICU admission. The rate of newborns with an APGAR score of less than 7, in both first and fifth second after birth, was significantly higher in the GA group, as well as the need for NICU admission.

**Conclusion:**

This study clearly emphasizes that the TTI are shortest when using GA or conversion of labor epidural analgesia to surgical anesthesia. Meanwhile, GA is also linked to higher rates of admissions to ICU as well as poorer neonatal outcomes compared to the other groups. Additionally, our study uncovered a low rate of GA, and relatively low rate of regional anesthesia failure, which meets the accepted standards.

## Introduction

In the last years, we see a dramatic increase in the rate of elective cesarean deliveries (CD). In England, for instance, the rate of initial CD labors rose from 11% to 16% in a 10 year period,[1] and likewise in the rate of CD in general (30% of labors in England[1] and 33% in the United States[2]). In some counties, CD even constitutes the majority of labors (around 55.5% in Brazil and in Egypt).[2]

The reasons and indications for the performance of CD may be sorted in a few different manners. The first is by distinction between maternal indications, such as preeclampsia, hemorrhage, high blood pressure, and past cesarean sections; fetal indications, such as macrosomia, abnormal fetal presentation, fetal distress and multiple gestation, and other obstetric reasons such as arrest of descent, placenta previa, cord prolapse, maternal preference, and so on.^[3, 4, 5]^ These very reasons may also be divided and separated on the basis of urgency of the operation for saving the life of the mother and/or newborn, or for the improvement of other outcomes of labor, for example, herpes prevention. Hence, the importance of surgery safety increases even more, as we choose CD to avoid neonatal herpes, for example, aiming to choose the safest path. Ensuring the maternal and fetal safety is the main anesthetic goal in CD’s, and in order to enable its achievement, multidisciplinary coordination, and collaboration are crucial. In order to create better communication between the anesthesiologists and gynecologists, the NICE organizations developed a classification of cesarean section urgency,[6,7] which was recently adopted by the Royal College of Obstetricians and Gynaecologists (RCOG) and the Royal College of Anaesthetists (RCoA).[8] As in any surgery, the anesthesiologist’s responsibility is to ensure the patient’s safety. In CD, there is great importance to matching the type of anesthesia with the urgency of the surgery and the maternal and fetal medical condition. Regional Anesthesia (RA) is generally preferable,[6,9] though in critical cases, GA is often necessary.

There are some solid reasons for the preference of RA over general anesthesia (GA).[10] The main reason is that maternal mortality rates in cases with GA are more than twice as high[11] (and even up to 16 times as high in certain case series).[12] Moreover, there is the concern of difficult intubations.[13,14] Accidental Awareness during General Anesthesia (AAGA),[15] the risks involved in the transfer of anesthetic medication from maternal to fetal blood,[16] and more. Besides the urgency, there are other indications for GA such as the patient’s refusal to RA, failure of RA,[17] coagulopathy,^[18, 19, 20]^ hemodynamic instability and increased intracranial pressure. Another factor that may make GA preferable is the availability and timeframe of the anesthesia. The time from making the decision to operate until reaching adequate anesthesia is significantly shorter in GA as compared to RA.^[12,21, 22]^ It should also be noted that the type of anesthesia chosen will have an effect on blood loss and on the need to use blood products.[23,24] Of all emergency CD’s, the rate of GA usage in accordance with the urgency is around 7–15%.^[10,25, 26]^ Some case series even present a rate as high as 50% in certain cases.[27,28]

## Materials and methods

In this retrospective cohort study, we focused on emergency CD performed in Shaare Zedek Medical Center, comparing the ones performed with GA to the ones performed using RA. We compared the times elapsed from the patient’s admission to the operation room until the beginning of surgery (time to incision-TTI) and from admission to operation room until delivery (time to delivery - TTD); maternal outcomes and complications, such as the need for blood products and transfer to ICU; and neonatal outcomes and complications, such as PH, APGAR, and transfer to NICU.

We have gathered data regarding emergency CD performed between January–December 2018. The data was digitally extracted from automated anesthesia records by the two coauthors (KW & RJ). The surgeries were identified as CD according to the diagnosis code, and as emergent according to a specific system mark. From these cases, additional data regarding the patients and the newborns was extracted and embedded into Excel tables. Any missing data was manually gathered from the medical records. Finally, all data was cross-referenced in order to eliminate clerical errors. The study got the approval of the institutional ethics committee (Number ID-RCB: 0062-19-SZMC).

The inclusion criteria were emergency CD performed in 2018, exclusion criteria were, as is customary,[29] multiple gestation, cases with IUFD, and alongside cases in which the anesthetic protocols were not digital or unavailable.

The primary outcome of the study was the time to incision (TTI) and time to delivery (TTD). The secondary outcomes were the maternal complications, such as the need for blood products and transfer to ICU; need for intraoperative supplemental intravenous analgesia/sedation in the different groups of neuraxial anesthesia (NAA) and neonatal outcomes and complications, such as PH, first, and fifth minute APGAR score and transfer to NICU. Additional recorded data was the patient’s preoperative data (age, number of pregnancies, number of labors, and number of past cesarean sections) and additional monitoring.

## Results

During the follow up period, 1253 patients underwent emergency CD. After exclusion according to the criteria, 1059 patients were included in the study. The tables presented below only include the data of 1025 patients for which the anesthesia type was not changed during surgery. The groups’ characteristics are presented in Table 1.

**Table 1:** Preoperative Characteristics

	GA	Epidural	Spinal	CSE
**Count**	79	383	547	16
**Mother’s age, Years**	31.41 ± 6.76 (19–45)	28.98 ± 6.11 (16–49)	31.83 ± 6.37 (19–52)	32.75 ± 7.61 (20–46)
**Parity**	4.59 ± 3.52 (1–17)	2.99 ± 2.94 (1–18)	4.43 ± 3.28 (1–19)	4.31 ± 2.44 (1–8)
**Past CS**	0.59 ± 1.10 (0–5)	0.20 ± 0.41 (0–2)	0.73 ± 1.03 (0–6)	1.69 ± 1.62 (0–5)
**Gestational Age, Weeks**	36.76 ± 4.26 (26–41)	39.55 ± 1.71 (29–43)	37.53 ± 3.20 (25–42)	37.25 ± 3.02 (30–41)

Values are given as Mean ± SD (range); GA - General Anesthesia; CSE - Combined Spinal Epidural

The rate of GA amongst all patients was 7.7%. The correlation between the anesthesia type and the maternal outcomes is presented in Table 2.

**Table 2:** Maternal Outcomes

	GA	Epidural	Spinal	CSE
**TTI, Min**	11.19 ± 10.74 (0.23–48.48)*	11.29 ± 5.64 (0.68–38.27) NS	19.41 ± 7.77 (0–60.9)	29.94 ± 9.06 (10.22–43.57)
**TTD, Min**	14.15 ± 11.93 (2.00–53.02)*	16.17 ± 7.55 (2.17–78.77) NS	25.46 ± 9.99 (1–97)	39.09 ± 12.09 (14.22–61.72)
**Blood products**	9 (11.4%)*	2 (0.5%)	4 (0.7%)	1 (6.3%)
**ICU admission**	5 (6.3%)*	0 (0%)	1 (0.2%)	0 (0%)

Values are given as Mean ± SD (range) or as number (percent); GA - General Anesthesia; CSE - Combined Spinal Epidural; TTI - Time To Incision; TTD - Time To Delivery; NS - not significant compared to GA; * p < 0.05 compared to other groups.

GA is, as seen above, correlated with shorter TTI and TTD, but also with greater use of blood products and need for ICU admission. In our experimental group, there were 2 (1.9%) cases in which the intubation was labeled “difficult” by the anesthesiologist. In both these cases, video laryngoscope was successfully used for intubation.

Intraoperative supplemental intravenous analgesia/sedation, defined in this study as using of one or more of the following medications: Fentanyl 50 mcg, Ketamine 12.5 mg, Midazolam 2 mg, Propofol 50 mg in the different groups of NAA was 15.9–37.5%.

27.4% patients in the Epidural group required sedation. No events of aspiration or other sedation-related complications had been recorded. In our group of patients, of the 403 who started with epidural in the delivery room, 20 (4.9%) required conversion of the anesthesia type, of which 9 (2.2%) were converted to spinal following a failed epidural, 7 (1.7%) were converted to GA following a failed epidural, and 4 (1%) were converted to GA, in spite of a proper epidural, due to the urgency of the operation. From 561 patients who started with spinal, 14 (2.49%) required conversion to GA following a failed spinal or additional intraoperative complication. The rate of sedation in Spinal group was 15.9%.

The correlation between the type of anesthesia and the neonatal outcomes is presented in Table 3.

**Table 3:** Neonatal Outcomes

	GA	Epidural	Spinal	CSE
**APGAR1 < 7**	38 (48.1%)*	63 (16.4%)	87 (15.9%)	3 (18.8%)
**APGAR5 < 7**	19 (24.1%)*	14 (3.7%)	23 (4.2%)	0 (0%)
**Birth Weight**	2825 ± 870 (700–4180)	3311 ± 548 (1126–4652)*	2970 ± 783 (570–5114)	2786 ± 667 (1210–3958)
**Transfer to NICU**	28 (35.4%)*	37 (9.7%)	122 (22.3%)	7 (43.8%)
**PH**	7.205 ± 0.153 (6.71–7.405)**	7.256 ± 0.094 (6.776–7.442)	7.271 ± 0.975 (6.894–7.506)**	7.22 ± 0.136 (6.895–7.391)

Values are given as Mean ± SD (range) or as number (percent); GA - General Anesthesia; CSE - Combined Spinal Epidural; NICU - Neonatal Intensive Care Unit; * p < 0.001 compared to other groups. ** p < 0.05 between those two groups.

## Discussion

Our study’s main objective was to find whether there is a statistically significant variance in TTI and TTD, depending on the different types of anesthesia used in emergency CD; GA and various NAA types.

Analyzing the results, we found no clinically substantial difference between the groups in terms of demographic parameters. Moreover, there was no significant difference in TTI when comparing the groups of patients who received GA for CD and those who were converted from labor epidural analgesia to surgical epidural anesthesia for CD (epidural conversion). TTD times of these groups also did not differ significantly. While the fact that GA provides the shortest operation-times is a consensus in literature, the reality that epidural conversion allows similarly short times is not as consistent.[22,30]

The use of spinal anesthesia for CD is common in the absence of labor epidural analgesia,[28] though according to the data we have uncovered, there is a TTI difference of more than 8 minutes in spinal anesthesia group compared to GA or epidural groups.

In our hospital, the use of combined spinal epidural (CSE) is not very common, and according to the data gathered, it prolongs the TTI by almost 3-fold as compared to GA or epidural conversion. Thus, the use of CSE may be a reasonable option only in non-critical cases.

As secondary outcomes, we examined data regarding the use of blood products, admission to ICU and the need for additional sedation on top of NAA. It is notable that in the GA group, the need for blood products was substantially greater (11.4% of patients in this group received blood products, and 6.3% of them were even admitted to ICU, mainly due to massive bleeding). This finding matches reports in literature,[23,24] As for the need of additional sedation, the group of patients who got epidural conversion needed additional sedation at far higher rates, as could be expected.[28] It should be noted that both in the epidural group and in the spinal group, in which 15.9% were sedated, no unusual events of aspiration or other sedation-related complications had been recorded, and there were no admissions to ICU.

One of the common aspects referenced in relation to emergency CS anesthesia, is the rate of NAA failure.[28] In our group of patients, of the 403 who started with epidural in the delivery room, only 20 (4.9%) required conversion of the anesthesia type, of which 9 (2.2%) were converted to spinal following a failed epidural, 7 (1.7%) were converted to GA following a failed epidural and 4 (1%) were converted to GA, in spite of a proper epidural, due to the urgency of the operation.

According to the guidelines of the Royal College of Anesthetists, an RA to GA conversion rate of less than 5% in emergency CS is considered an indication for a high-quality obstetric anesthesia unit.[28] If so, our unit meets this standard. Expectably, there have also been cases of failed spinals; of the 561 patients who started off with spinal, 14 (2.49%) required conversion to GA following a failed spinal. Nevertheless, this rate also meets the guidelines specified above.

As previously noted, one of the reasons for avoiding GA in obstetrics, as much as possible, is the relatively high risk of difficult intubation and its complications. In our experimental group, there were 2 (1.9%) cases in which the intubation was labeled “difficult” by the anesthesiologist. In both these cases, video laryngoscope was used for intubation, and there were no further complications or need for ICU admission.

As for neonatal outcomes, there was a clear and significant correlation found between all examined outcomes and GA. APGAR1, APGAR5, and pH outcomes were all substantially poorer in the GA group. Furthermore, the rate of NICU admissions was significantly higher in GA group (35.4%) compared to epidural and spinal groups (9.7% and 22.3%, respectively). Nevertheless, it should be noted that the birth weights and gestational ages of the epidural group were also markedly better.

As limitations of this study, we can mark that it was a retrospective research, with data gathered from medical records, may inherently suffer from some inconsistencies; hence, we will shortly begin working on a similar study in a prospective cohort outline. Given the existing structure of the medical records, both the time of making the decision to operate and its levels of urgency are not precisely recorded. Therefore, we were not able to calculate the decision delivery time properly, nor categorize the operations in terms of urgency.

## Conclusion

This study clearly emphasizes that the times from admission to the operation room until incision are shortest when using GA or conversion of labor epidural analgesia to surgical anesthesia. Meanwhile, GA is also linked to higher rates of admissions to ICU as well as poorer neonatal outcomes compared to the other groups. Additionally, our study uncovered a low rate of GA, and a relatively low rate of RA failure, which meets the accepted standards.

Accordingly, it may be suggested that both maternal and neonatal outcomes are expected to be better in the epidural group. Moreover, this method enables the shortest TTI. Thus, it may be recommended to perform epidural analgesia in the early stages of labor, for all cases with a known high risk of reaching cesarean section delivery.

Every medical center works under unique conditions, with different population types, which undoubtedly affect the rates of GA and epidural analgesia use for labors. Therefore, we advise each medical center to examine its specific data and adjust its standards in accordance with the current guidelines found in literature.
